# Cognitive and behavioral approaches to occupational stress management: The case of adult education administrative workers in Nigeria

**DOI:** 10.1097/MD.0000000000036825

**Published:** 2024-01-26

**Authors:** Nkechi Anyadike, Happiness Kodichinma Ogiri, Solomon Uchenna Agbo, Bessong Napoleon Osang, Columbus Deku Bessong, Ozurumba Iheanyichukwu Godwin, Ogechi Nkemjika, Ekere Onyinye, Imo Charity Onyeodiri, Mary Okengwu, Linus Okechukwu Nwabuko, Mkpoikanke Sunday Otu, Vera Victor-Aigbodion, Roland Ndille

**Affiliations:** a Department of Public Administration and Local Government, University of Nigeria Nsukka, Nsukka, Nigeria; b Institute of African Studies, University of Nigeria Nsukka, Nsukka, Nigeria; c Department of Business Education, Faculty of Vocational Teacher Education University of Nigeria Nsukka, Nsukka, Nigeria; d Department of Continuing Education and Developmental Studies, University of Calabar, Calabar, Nigeria; e Department of Adult Education and Extra Mural Studies, University of Nigeria Nsukka, Nsukka, Nigeria; f Department of Educational Psychology, University of Johannesburg, Johannesburg, South Africa; g University of Buea, Buea, Southwest Region Cameroon.

**Keywords:** adult education workers, cognitive and behavioral approach, Enugu State, Nigeria, occupational stress, public administrative, staff members

## Abstract

**Background:**

Those in administrative positions in adult education are more likely to suffer from stress because of the hard work they do, long hours in the office, a lack of adequate medical and welfare packages, and a lack of financial aid. In this study, adult education workers in Nigeria were assessed on the effectiveness of a cognitive and behavioral approach to managing occupational stress in public administration.

**Method:**

This study was a group-randomized trial in which 94 adult education workers occupy public administrative positions within Enugu State, Nigeria, participated. Data were collected using 2 instruments, the Perceived Stress Scale, and the Workplace Stress Scale, which were validated by cognitive and behavioral psychologists at the University of Nigeria, Nsukka. The instruments contained internal construct and content validity as determined by Cronbach alpha. ANCOVA was employed to test for hypotheses and answer research questions.

**Results:**

This study demonstrates that cognitive and behavioral approaches are significantly effective in managing occupational stress among adult education workers who work for public agencies.

**Conclusion:**

To improve stress management capacity among adults in public administration positions, researchers recommend frequent exposure to cognitive and behavioral approaches.

## 1. Introduction

In spite of technological advances, the issue of stress affects the productivity of public workers. Because of the demands of their occupations, many Nigerian workers suffer from high levels of administrative stress. The determinants of occupational stress, which is work-based, are the emotive, cognitive, and behavioral challenges people face at work and how they respond to them. In today workplace, workplace stress has been described as an inevitable phenomenon, not only in the workplace but also in all aspects of life.^[1–3]^ Stress affects both employees and administrators at an organization. In addition to the arduous nature of their work, the long hours they spend in the office, the lack of adequate medical and welfare packages, and poor financial assistance, public administrators are at greater risk of stress among the different groups of workers.^[4]^ A major cause of stress for workers at work is expecting more from administrative occupational staff when the support system is insufficient.^[5]^

A large proportion of the workspace is occupied by adult education workers. They administer assignments and duties, maintain the working system of a firm, and maintain orderliness, guidance, and uniformity in the operations of a business.^[6]^ Their responsibilities encompass every aspect of a firm or organization. Administrators are, however, exposed to intense stress levels as a result of their responsibilities, which adversely affects their health and overall performance. In addition to excessive workload, unclear job definitions, time constraints, operational fluctuations, and problems with relationships, public administration occupational stress can also be caused by several other factors.^[7,8]^

In today business world, and in a bid to meet targets, most administrators perform several responsibilities simultaneously. As a result, they are overburdened and stressed. Administrators are only being exposed to stressful situations when they are under pressure to complete assignments, perform organization tasks effectively, and meet family needs.^[9,10]^ In addition, stress can also arise when administrators do not grasp the task at hand properly. It occurs when tasks are unclear, administrators are not clear on a firm vision and prospects, or when a company changes its operations without proper planning and preparation. As a result of unclear job descriptions and unplanned changes in operations, workers may misperceive, be disappointed, be stressed, and eventually become frustrated.^[11,12]^ Time constraints are another cause of administrative occupational stress. According to research,^[13]^ administrators are always battling with time factors in the workplace. There is always a limited amount of time to accomplish humongous tasks. Administrators face a high level of stress because they are at the forefront of organization activities, trying to manage multiple work schedules within a constrained timeframe, directing the organization operations, and delivering efficiently on their specific assignments at the same time.^[14]^ It is also common for public administrators to experience relationship issues as stressors.^[15]^ The stress that administrators experience includes breakups in marriages and conflicts arising from daily interactions with different workers, including blackmailing and insubordination from subordinates and at home. Conflicts can arise from misunderstandings or exertions of authority among public administrators and their associates, superiors, and even family members. A result of this is emotional or behavioral problems that lead to stress among administrators.

There are several negative consequences of occupational stress in the public administrative sector for businesses. As well as reducing workers’ productivity, occupational stress also encourages work blunders and health complications.^[2]^ Public administrators face a number of stressors, including ill health, loss of joy and fulfillment in their jobs, underperformance, and burnout.^[16,17]^ Workplace stress can be harmful to administrators’ health, leading to high blood pressure, heart problems, weakened immune systems, and deterioration, anxiety, unrest, and depression.^[18,19]^ When administrators lose motivation to work, they have difficulty concentrating. Stress causes debilitation in addition to reducing workers’ motivation and enthusiasm. The result is a decrease in their productivity and ability to meet organizational goals.^[20]^ Stress is also a contributing factor to burnout among public administrators.^[21]^ Burnout is when public administrators feel persistently drained, mentally and physically, which results in indifference and pessimism in approaches, and leads to a loss of satisfaction in their work.

Because of its unhelpful effects on individuals from all walks of life, occupational stress has become a controversial topic in business circles. Public administrators in the business world face a growing need for interventions to lessen the sway of stress. Cognitive and behavioral approaches (CBA) are one such intervention. The cognitive and behavioral approach to stress management has received wide recognition in literature.^[4,22]^ Cognitive behavioral therapy (CBT) is the basis of CBA, which is premised on the idea that undesirable thought patterns and stimuli can negatively affect a person emotions and behavior in the workplace, and therefore must be identified and corrected. Individuals can change the way they think and relate to others by identifying adverse thinking patterns in them and replacing them with more acceptable and progressive thinking patterns. Therefore, CBT helps individuals develop strategies to help them survive, solve problems, and regulate their emotions.^[4]^

Among administrators, CBA is particularly important for managing and reducing occupational stress. Through CBA, individuals can manage stress in the workplace positively, develop a healthier perception of themselves and others, and increase their resilience for a more positive work environment by identifying cognitive biases such as catastrophizing, overgeneralizing, and damaging self-talk.^[23]^ CBA addresses problems arising from work-life balance, since imbalance in the work-life equation is one of the major occupational stressors for workers,^[24]^ since it equips people to manage and respond better to occupational stressors. Aside from equipping administrators with effective strategies for resolving relationship conflicts at work and at home, CBA assists in fostering a healthy work-life balance among public administrators and reducing their stress levels. The paper adopts CBA as an interventional package for managing public administrative occupational stress because finding a solution to stress is a burning subject requiring attention.

## 2. Methods

### 2.1. Obtaining ethical approval

The study complied with the Helsinki declaration and the standard research requirements of the University of Nigeria, Nsukka.

### 2.2. Design of the study

In this study, a group randomized pretest–post–test pattern was used. The pretest–post–test experimental design can provide ample evidence to support a causative explanation of data in a study. It determines the impact of an intervention on a particular characteristic. In pretest–post–testing, subjects can be randomized into 2 groups—a control and a treatment group—preventing researchers from prejudging which group participants belong to. As an experimental group, members receive intervention, while as a control group, they receive no intervention. A pretest—post-test study measures the presence of a trait twice—before treatment (pretest), and after treatment (posttest). In this study, we assess the effectiveness of a CBA intervention program in managing stress among public administration occupational staff. After administering the pretest to all members, the researchers randomly assigned participants to either a control group or a treatment group. Only the treatment group received the CBA intervention. After that, all members took the post-test. Both groups had the same pretest and post-test.

### 2.3. Participants in the study

Following a cluster sampling technique, the researchers registered 117 adult education workers in Enugu State, Nigeria who serve in public administrative positions. Among the inclusion criteria for the study were being a public administrator, having stress-related problems, a doctor report on the severity of the participant case, signing informed consent forms, and being screened for eligibility. A Perceived Stress Scale-Ten (PSS-10), developed by Cohen in 1983,^[25]^ and a Workplace Stress Scale (WSS) developed by Marlin Company and the American Institute of Stress in 2001^[26]^ to assess the frequency and severity of stress among workers^[27]^, were used to test eligibility for the study. The study exclusion criteria included being currently on a stress remedy program, having mental imbalance, or being in any stressful situation at the time of the study, such as marriage breakdown, death of dear ones, forfeiture of property, money, or demotion. Informed consent forms were signed by all participants to show that they were willing to participate. The participants' characteristics are presented in Table 1.

**Table 1:** Description of participants.

Gender	Intervention group	No-intervention group
Male	28 (29.79%)	21 (22.34%)
Female	19 (20.21%)	26 (27.66%)
Total	47 (50%)	47 (50%)

### 2.4. The measures

For testing the severity of stress among public administrative occupational staff members, the researchers used the PSS-10 and the WSS.

### 2.5. Perceived Stress Scale

PSS-10 was developed by Cohen^[25]^ and contains 10 items. Using PSS-10, workers were asked to provide responses to questions regarding the frequency of their stressor thoughts and feelings within the past 30 days, which was designed to estimate the severity of stress. PSS-10 questions are scored on a 5-point scale of 0 = never, 1 = almost never, 2 = sometimes, 3 = often, and 4 = very often. The scale is divided into 4 parts. 0 is rated 0, 1 = 1, 2 = 2, 3 = 3, and 4 = 4.^[28–34]^

### 2.6. Workplace Stress Scale

In order to measure the severity and frequency of workplace stressors among workers, the Marlin Company and American Institute of Stress developed the WSS. The WSS comprises 8 items that prioritize the feelings of respondents toward their occupations.^[35]^ Statements on the WSS are graded on a 5-point scale of never = 1, rarely = 2, sometimes = 3, often = 4, and very often = 5.

### 2.7. The procedure

Public administrators were invited to join the study voluntarily, after the researchers advertised it and informed all willing participants of the study rationale. Participants were also given the liberty to withdraw from the program if dissatisfied. However, they had to provide informed consents to participate in the study first. Five experts in stress management and CBT implementation assisted the researchers in executing the research. All participants were public administrators with various levels of stress-related problems. Research assistants in this study were professionals, so the researchers met with them 2 times a week for only 2 weeks to explain the purpose of the study and the techniques used to administer the CBA interventional program.

After conducting a pretest for reference purposes, researchers randomly assigned participants (94) to intervention (47) or nonintervention (47) groups. A CBA intervention was given to the treatment group over a period of 6 weeks, spread out across 12 sessions—2 sessions per week—while the nonintervention group received no intervention. The intervention exercise lasted 50 minutes per session for all participants in the intervention group. A post-test was administered to the 2 groups by the researchers after 6 weeks of the intervention. A 2-session follow-up encounter took place 2 months after the intervention, during which all participants had their test repeated. In a previous study, a modified CBT intervention program was used and proved effective for public administrators suffering from stress-related issues. This follow-up test was designed to make a final case for CBT interventional programs.

### 2.8. The intervention

#### 2.8.1. The cognitive behavioral approach to stress management.

The CBA is an interventional package that focuses on bringing adjustment to people behavior and cognitive processes, as well as reducing the signs of various disorders in people thinking patterns.^[36,37]^ Using CBT intervention strategies, behavior stimulation, mental reorganization, recreation exercises, problem solving, boldness, and communication training are among the tactics.^[37]^ The intervention program for the present study aimed to help public administrators manage their thoughts and modify their behavior in any way possible to avoid getting trapped amidst stressors through CBA for stress management. As part of the intervention package, participants were exposed to self-overwhelming, dysfunctional, and disparaging thoughts harmful to their psychological, occupational, and social wellbeing, and taught how to handle and manage stressors at work.^[38]^

As part of the CBA intervention, participants were taught rational thinking, stress-resolution skills, logical reasoning, appreciation, objective assessment of situations, fact evaluation, blending, reflection, recreation, distending, and mental reshuffle techniques. A CBA package was used in this study to help participants think, recognize stress-stimulating thoughts, and assess these thoughts objectively. A CBA intervention was proposed in this study to correct dysfunctional workplace impressions such as “I must have continuous support from colleagues and superiors,” or “affairs in the workplace must follow a predictable path,” adapted from literature.^[4]^ As part of the CBA intervention, the researchers planned to provide active sessions where participants would experience the side effects of misconceived ideas, receive guidance on analyzing arguments, making inferences, evaluating issues, and ultimately, be able to manage stressors around them by making informed choices.

### 2.9. Analyses of the data

With a probability level of 0.05, the study is statistically significant. Both the intervention and nonintervention groups were able to examine data from continuous variables (e.g., age and stress levels) using average scores (M) and standard deviations (SD). During testing for any discrepancies between the mean scores of the continuous variables between the experimental and nonintervention groups, an analysis of variance (ANOVA) was used for repeated measures of values among a sample. The ANOVA for repeated values is used to test the effectiveness of an intervention over 3 discrete time periods on individuals.

In the current study, the researchers examined the impact of a CBA interventional program on managing stress among public administrators at 3 different times, namely, before intervention (pretest), after intervention (post-test), and 2 months after intervention (follow-up). After administering the instruments, a *t* test was used to determine whether the mean scores of the continuous variables between treatment and nonintervention groups differed at 3 different time points in the study. The authors further computed the deviations of each of the items according to the scores collected at the different periods. SPSS 23 (IBM Corp, Armonk, NY) provided useful assistance in the analysis process. It was the Partial Eta Squared that tested the success of the CBT interventional package on the dependent variables that provided answers to the research questions.

## 3. Results

In Table 2, the cognitive and behavioral approach (CBA) is summarized in terms of its effect on public administrative workers who suffer from occupational stress. During the pretest, there was no significant baseline difference between the mean scores and SD of public administrative workers who were exposed to the CBA treatment and those who were exposed to the usual care treatment, with mean scores and SD of 30.8696 ± 5.51609 and 30.0200 ± 4.83394 respectively. According to the results, public administration staff had high mean scores across the 2 groups, indicating high occupational stress levels.

**Table 2:** Descriptive statistics showing the mean scores of PSS-10 among public administrative workers with occupational stress.

		N	Mean	Std. deviation	Std. error	95% confidence interval for mean	
						Lower bound	Upper bound
PSS pretest	Treatment group	46	30.8696	5.51607	0.81330	29.2315	32.5076
	No-treatment control group	50	30.0200	4.83394	0.68362	28.6462	31.3938
	Total	96	30.4271	5.16210	0.52685	29.3811	31.4730
PSS post-test	Treatment group	46	9.7174	1.75958	0.25944	9.1949	10.2399
	No-treatment control group	50	32.2600	5.80925	0.82155	30.6090	33.9110
	Total	96	21.4583	12.12559	1.23756	19.0015	23.9152
PSS follow-up	Treatment group	46	6.9348	1.65196	0.24357	6.4442	7.4254
	No-treatment control group	50	31.2200	5.24225	0.74137	29.7302	32.7098
	Total	96	19.5833	12.81419	1.30784	16.9869	22.1797

PSS = Perceived Stress Scale.

As shown in Table 2, the post-test mean scores and SD of public administrative staff in the treatment and no-treatment groups are respectively 9.7174 ± 1.75958 and 32.2600 ± 5.80925. According to the results, public administrative staff subjected to CBA had low mean scores and SD, while public administrative staff subjected to usual-care had high mean scores and SD, demonstrating that CBA reduced occupational stress among public administrative staff effectively.

The mean scores and SD of public administrative staff exposed to the CBA intervention and those exposed to usual care treatment were 6.9348 × 1.65196 and 31.2200 × 5.24225, respectively. In the CBA treatment group, the mean score and SD are low, while in the usual-care group, the score and SD are high. According to the results, the CBA was effective in managing stress among public administrative staff at the post-test.

It is evident from Table 3 that at post-test and follow-up, there was a significant difference in occupational stress between public administrative staff exposed to CBA treatment and those exposed to usual-care treatment [F(1,94) = 638.301, *P* = .000] and follow-up [F(1,94) = 903.926, *P* = .000]. Accordingly, the hypothesis stating that occupational stress between public administrators exposed to CBA intervention and those exposed to usual-care treatment is not significant was rejected. Consequently, compared with usual care, the CBA package significantly reduced occupational stress experienced by public administrative staff.

**Table 3:** ANOVA showing the significance of CBA for public administrative occupational stress management measured by PSS-10.

		Sum of squares	df	Mean square	F	Sig.
PSS pretest	Between groups	17.292	1	17.292	0.647	0.423
	Within groups	2514.197	94	26.747		
	Total	2531.490	95			
PSS post-test	Between groups	12,174.887	1	12,174.887	638.301	0.000
	Within groups	1792.946	94	19.074		
	Total	13,967.833	95			
PSS follow-up	Between groups	14,129.949	1	14,129.949	903.926	0.000
	Within groups	1469.384	94	15.632		
	Total	15,599.333	95			

ANOVA = analysis of variance, CBA = cognitive behavioral approach, PSS = Perceived Stress Scale.

**Figure 1.**
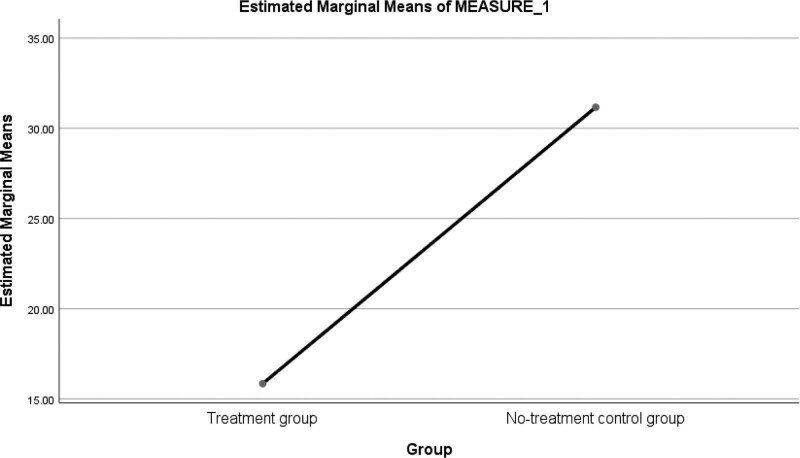
Line graph showing the mean response on PSS-10 across treatment and no-treatment groups. PSS = Perceived Stress Scale.

PSS-10 response graphical representations across treatment and control groups are shown in Figure 1.

According to the WSS, Table 4 summarizes the effects of the cognitive and behavioral approach (CBA) on public administrative workers suffering from occupational stress. According to the table, there were no significant differences at the pretest between public administrative workers exposed to the CBA treatment and those exposed to usual care treatment in terms of mean scores and SD with 29.3913 ± 6.04604 and 29.9200 ± 4.8480 respectively. In the pretest, high mean scores were found in both groups, indicating a high level of occupational stress.

**Table 4:** Descriptive statistics showing the mean scores of WSS among public administrative workers with occupational stress.

		N	Mean	Std. deviation	Std. error	95% confidence interval for mean	
						Lower bound	Upper bound
WSS pretest	Treatment group	46	29.3913	6.04604	0.89144	27.5959	31.1868
	No-treatment control group	50	29.9200	4.84806	0.68562	28.5422	31.2978
	Total	96	29.6667	5.43220	0.55442	28.5660	30.7673
WSS post-test	Treatment group	46	10.1957	2.09358	0.30868	9.5739	10.8174
	No-treatment control group	50	29.0000	3.13636	0.44355	28.1087	29.8913
	Total	96	19.9896	9.81459	1.00170	18.0010	21.9782
WSS follow-up	Treatment group	46	7.8913	1.58083	0.23308	7.4219	8.3608
	No-treatment control group	50	31.1600	3.37070	0.47669	30.2021	32.1179
	Total	96	20.0104	11.98288	1.22300	17.5825	22.4384

WSS = Workplace Stress Scale.

Additionally, the respective mean scores and SD of public administrative staff suffering from occupational stress in the treatment group and no-treatment group at the post-test are 10.1957 ± 2.09358 and 29.0000 ± 3.13636. A low mean score and SD were observed for public administrative staff treated with CBA, but a high mean score and SD were observed for public administrative staff treated with usual care. Accordingly, the CBA treatment was effective in reducing occupational stress among public administrators.

**Figure 2.**
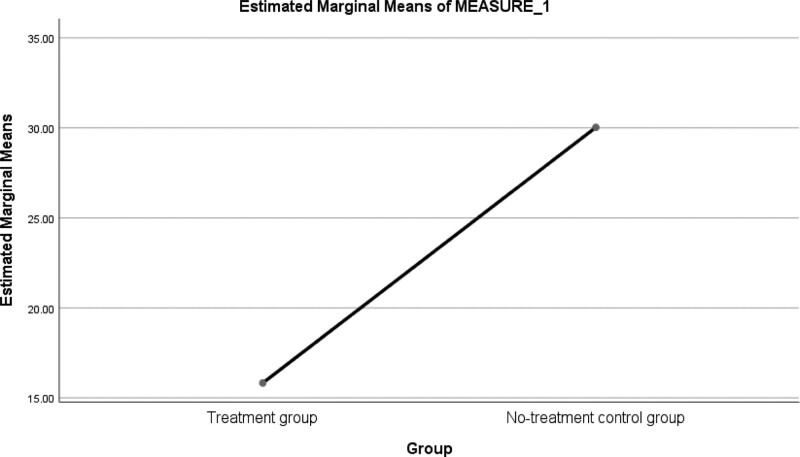
Mean response on WSS across treatment and no-treatment groups. WSS = Workplace Stress Scale.

A follow-up analysis revealed that the mean scores and SD of public administrative staff exposed to the CBA intervention and those exposed to usual care treatment were 7.8913 ± 1.58083 and 31.1600 ± 3.37070 respectively. The results show that public administrative staff exposed to CBA treatment had lower mean scores and SD than staff given only usual-care treatment. At the follow-up as well as at the post-test, CBA reduced occupational stress among public administrative staff effectively. The researchers tested hypothesis 2 at a level of significance of 0.05 in order to strengthen research question 2. As shown in Figure 2, the WSS for the 2 groups is plotted on a line graph.

In Table 5, the ANOVA results indicate that, when occupational stress was measured with WSS, there was a significant difference between those exposed to CBA treatment and those exposed to usual-care treatment [F(1,94) = 1172.407, *P* = .000] and follow-up [F(1,94) = 1822.166, *P* = .000]. Therefore, the hypothesis that stated that there was no significant difference in occupational stress between public administrative staff exposed to CBA treatment and public administrative staff exposed to the usual-care treatment was rejected, which means that the CBA treatment was effective in reducing occupational stress among public administrative staff.

**Table 5:** ANOVA showing the significance of CBA for public administrative occupational stress management measured by WSS.

		Sum of squares	df	Mean square	F	Sig.
WSS pretest	Between groups	6.697	1	6.697	0.225	0.636
	Within groups	2796.637	94	29.751		
	Total	2803.333	95			
WSS post-test	Between groups	8471.750	1	8471.750	1172.407	0.000
	Within groups	679.239	94	7.226		
	Total	9150.990	95			
WSS follow-up	Between groups	12,971.813	1	12,971.813	1822.166	0.000
	Within groups	669.177	94	7.119		
	Total	13,640.990	95			

ANOVA = analysis of variance, CBA = cognitive behavioral approach, WSS = Workplace Stress Scale.

### 3.1. Findings discussion

It was found that at both the post-test and follow-up exercises, there was a significant difference in occupational stress between public administrative staff exposed to the cognitive and behavioral approach and those exposed to the usual care treatment. Thus, a cognitive behavioral program is effective in reducing occupational stress among public administrative staff, and that reduction was sustained after 2 months of follow-up. Public administrative staff who were exposed to the CBA program in the study were able to control occupational stressors. Researchers support Ellis’ theory that cognitive behavioral approaches (CBAs) can help workers manage and reduce stress by identifying negative thought patterns and correcting them.^[4,22,39]^

Also, the findings of this study are consistent with the findings of previous studies that demonstrate that CBAs are effective in managing and reducing depression, anxiety, and stress, as well as improving a person health status.^[4,38,40–42]^ As found in the study, CBAs can be effective in changing dysfunctional thoughts and managing occupational stressors in science and social science education facilitators,^[43]^ which is in line with a similar study conducted in 2020 in Nigeria South-South region. Thus, exposing public administrative workers to the rudiments of the CBA to identify and manage occupational stress cannot be overstated.

### 3.2. Final thoughts

The CBA has been shown to be an effective method of reducing occupational stress in public administrators, so efforts need to be made to implement the CBA across Nigerian public administrative staff. The benefits of this approach will consist of reducing occupational stress among public administrators, boosting their productivity, and maintaining their health-physically, mentally, and emotionally.

## Author contributions

**Conceptualization:** Nkechi Anyadike, Happiness Kodichinma Ogiri, Solomon Uchenna Agbo, Bessong Napoleon Osang, Ozurumba Iheanyichukwu Godwin, Ogechi Nkemjika, Ekere Onyinye, Imo Charity Onyeodiri, Mary Okengwu, Linus Okechukwu Nwabuko, Mkpoikanke Sunday Otu, Vera Victor-Aigbodion, Roland Ndille.

**Data curation:** Nkechi Anyadike, Happiness Kodichinma Ogiri, Solomon Uchenna Agbo, Bessong Napoleon Osang, Columbus Deku Bessong, Ozurumba Iheanyichukwu Godwin, Ogechi Nkemjika, Ekere Onyinye, Mary Okengwu, Linus Okechukwu Nwabuko, Mkpoikanke Sunday Otu, Roland Ndille.

**Formal analysis:** Nkechi Anyadike, Happiness Kodichinma Ogiri, Solomon Uchenna Agbo, Bessong Napoleon Osang, Columbus Deku Bessong, Ozurumba Iheanyichukwu Godwin, Imo Charity Onyeodiri, Mary Okengwu, Linus Okechukwu Nwabuko, Mkpoikanke Sunday Otu, Vera Victor-Aigbodion.

**Funding acquisition:** Nkechi Anyadike, Bessong Napoleon Osang, Ekere Onyinye, Imo Charity Onyeodiri, Linus Okechukwu Nwabuko, Roland Ndille.

**Investigation:** Nkechi Anyadike, Happiness Kodichinma Ogiri, Solomon Uchenna Agbo, Columbus Deku Bessong, Ozurumba Iheanyichukwu Godwin, Ogechi Nkemjika, Ekere Onyinye, Imo Charity Onyeodiri, Mary Okengwu, Linus Okechukwu Nwabuko, Vera Victor-Aigbodion.

**Methodology:** Nkechi Anyadike, Happiness Kodichinma Ogiri, Solomon Uchenna Agbo, Bessong Napoleon Osang, Columbus Deku Bessong, Ozurumba Iheanyichukwu Godwin, Ogechi Nkemjika, Ekere Onyinye, Mary Okengwu, Linus Okechukwu Nwabuko, Mkpoikanke Sunday Otu, Vera Victor-Aigbodion, Roland Ndille.

**Project administration:** Bessong Napoleon Osang, Columbus Deku Bessong, Imo Charity Onyeodiri, Mkpoikanke Sunday Otu, Vera Victor-Aigbodion, Roland Ndille.

**Resources:** Happiness Kodichinma Ogiri, Ogechi Nkemjika, Imo Charity Onyeodiri, Mkpoikanke Sunday Out.

**Software:** Ekere Onyinye, Linus Okechukwu Nwabuko.

**Supervision:** Solomon Uchenna Agbo, Columbus Deku Bessong, Ozurumba Iheanyichukwu Godwin, Ogechi Nkemjika, Imo Charity Onyeodiri, Mary Okengwu, Linus Okechukwu Nwabuko, Mkpoikanke Sunday Otu, Vera Victor-Aigbodion.

**Validation:** Nkechi Anyadike, Happiness Kodichinma Ogiri, Solomon Uchenna Agbo, Ozurumba Iheanyichukwu Godwin, Mary Okengwu, Vera Victor-Aigbodion.

**Visualization:** Roland Ndille.

**Writing – original draft:** Nkechi Anyadike, Happiness Kodichinma Ogiri, Solomon Uchenna Agbo, Columbus Deku Bessong, Ogechi Nkemjika, Mary Okengwu, Mkpoikanke Sunday Otu, Vera Victor-Aigbodion, Roland Ndille.

**Writing – review & editing:** Nkechi Anyadike, Happiness Kodichinma Ogiri, Solomon Uchenna Agbo, Columbus Deku Bessong, Ozurumba Iheanyichukwu Godwin, Ogechi Nkemjika, Mary Okengwu, Mkpoikanke Sunday Otu, Vera Victor-Aigbodion, Roland Ndille.
